# Malaria and Hiv in Adults: when the Parasite Runs into the Virus

**DOI:** 10.4084/MJHID.2012.032

**Published:** 2012-05-07

**Authors:** Emanuele Focà, Silvia Odolini, Nigritella Brianese, Giampiero Carosi

**Affiliations:** Institute for Infectious and Tropical Diseases, University of Brescia. Brescia, Italy

## Abstract

Malaria and HIV/AIDS are among the principal causes of morbidity and mortality worldwide, particularly in resource-limited settings such as sub-Saharan Africa. Despite the international community’s efforts to reduce incidence and prevalence of these diseases, they remain a global public health problem. Clinical manifestations of malaria may be more severe in HIV infected patients, which have higher risks of severe malaria and malaria related death. Co-infected pregnant women, children and international travelers from non-malaria endemic countries are at higher risk of clinical complications. However, there is a paucity and conflicting data regarding malaria and HIV co-infection, particularly on how HIV infection can modify the response to antimalarial drugs and about drug-interactions between antiretroviral agents and artemisinin-based combined regimens. Moreover, consulting HIV-infected international travelers and physicians specialized in HIV care and travel medicine should prescribe an adequate chemoprophylaxis in patients travelling towards malaria endemic areas and pay attention on interactions between antiretrovirals and antimalarial prophylaxis drugs in order to prevent clinical complications of this co-infection.

This review aims to evaluate the available international literature on malaria and HIV co-infection in adults providing a critical comprehensive review of nowadays knowledge.

## Introduction

Malaria is one of the most important causes of morbidity and mortality in tropical regions, especially in sub-Saharan Africa and South-East Asia.^1^ The disease is caused by an infection sustained by a parasite of the genus *Plasmodium*; five species of *Plasmodium* (*P. falciparum, P. vivax, P. ovale, P. malariae* and *P. knowlesi*) can infect human and among these species, *P. falciparum* infection may be fatal.^2^

According to the *World Malaria Report 2010*, there were 225 million cases of malaria and an estimated 781 000 deaths in 2009. Most deaths occur among children living in Africa where a child dies every 45 seconds for malaria and this disease accounts for approximately 20% of all childhood deaths.^2^

Human Immunodeficiency Virus (HIV) is a retrovirus that can infect immune competent cells causing an impairment of host defense. After the introduction of Highly Active Antiretroviral Therapy (HAART) in 1996, the epidemiology of HIV infection has changed and clinical evolution turned a fatal disease in a treatable chronic infection with an improved quality of life and a reduction of morbidity and mortality.^3^ However, nowadays there are still alarming figures of HIV/AIDS infected people worldwide: in 2010 it was estimated that people living with HIV infection were 34 million, 2.7 million people became newly infected in the same year and AIDS-related deaths were 1.8 million, including 250 000 children. Two thirds of HIV infections were in sub-Saharan Africa.^4^ Therefore, there is a critical overlap between the two infections especially in sub-Saharan Africa resulting in particular concern for Public Health. The increasing number of people potentially co-infected makes this topic of particular interest in order to correctly understand and control both infections and their particular interactions.^5^

Clinical presentation of malaria in HIV-infected people, even if partially immune to *Plasmodium*, may be more severe and, although malaria is not the main cause of death among HIV-infected patients,^6^ in a systematic review of HIV-1 infection in Africa, malaria was identified as the third cause of HIV-related morbidity.^7^ This is more evident in special populations such as HIV-infected pregnant women,^8^ as well as in adults male and female with an severely impaired immune system^9^ and in HIV-infected travelers.^10^

Moreover, despite the importance of proper treatments against both infections, there is a lack of data regarding treatment issue: conflicting data are presented in literature about the effects of antimalarial drugs (for treatment and chemoprophylaxis) on antiretroviral agents and *vice-versa* and data about drug-interactions between antimalarial (especially artemisinin-based combinated treatment – ACT) and antiretroviral drugs are missing.^11^

Purpose of this article is to review the current knowledge on clinical tools of malaria and HIV co-infection in adults, focusing on pregnant women and international travellers, and to explore the interactions between antiretrovirals and antimalarial drugs, suggesting future research priorities.

## Interactions Between Malaria and HIV Infection

Sub-Saharan Africa represents the region most heavily affected by both malaria and HIV. In this setting, the overlap of these two infections is common and it would be important to understand their interactions and their correct management in order to limit their clinical burden.^12^

The influences between HIV and malaria are bidirectional and synergistic,^5^,^13^ and the negative effects of this co-infection, for the most part, seems to be due to immunological interactions: HIV replication impairs immune system and consequently malaria control;^14^ on the other hand, malaria itself enhances HIV replication by cytokines release and T-cell activation.^15^

Looking at HIV-infection during malaria, it seems that HIV infection worsens the capability to control parasitaemia because of deterioration of immune responses to malaria parasites; several studies reported an association between HIV infection and higher levels of malaria parasitaemia. Whitworth et al. demonstrated that higher levels of parasitaemia were more frequently detected among HIV-positive patients compared to those mono-infected (11.8% vs 6.3%, p<0.0001), moreover they found an association between lower CD4+ T-cell count and higher levels of parasitaemia (p=0.0076), and lower CD4+ T-cell count and risk of clinical manifestations of malaria.^14^ French et al. confirmed this data finding that incidence rates of *P. falciparum* malarial fever were indirectly proportional to CD4+ T-cell count,^16^ while Patnaik and colleagues supported the relationship between CD4+ T cell count and malaria parasitaemia in HIV-seropositive subjects finding an adjusted hazard ratio of 1.8 for a first parasitaemia episode, and of 2.5 for a second parasitaemia episode.^17^

The intensity of malaria transmission and the population’s level of acquired immunity may influence the clinical impact of malaria. Two major levels of malaria endemicity are described: i) areas of high (stable) malaria transmission, where most of adults have developed enough immunity that lead to poor clinical manifestations of malaria infection and where the reported incidence of malaria was ≥ 1/1000 per year in 2009;^4^ ii) areas of low (unstable) malaria transmission, where people have not acquired a significant level of immunity with consequent high level of clinical appearance of the infection. In these areas the transmission is more often seasonal and the reported incidence of malaria was < 1/1000 population per year in 2009.^4^ Severe malaria and consequent deaths seem to be higher in unstable transmission areas,^18^ but some studies have demonstrated an increase in malaria incidence in regions of stable malaria transmission when it is associated with HIV-infection.^14^,^16^ Whitworth’s study, previously mentioned, was conducted in malaria endemic regions in Uganda, and demonstrated that risk of clinical malaria was 4.0% among HIV-1-positive patients and 1.9% among HIV-negative patients;^14^ also French’s trial was conducted among people from Uganda. However, some studies show conflicting data not describing an increase in severity of malaria from areas of stable transmission. Cohen et al. found a statistical significant correlation (p=0.003) between severe malaria and HIV infection among people nonimmune to malaria, instead this association was not found in semi-immune patients (p=0.284).^9^ Other studies^19^,^20^ concluded for the lack of association between HIV infection and malaria outcomes, but they suffer form some limitations: patients analysed were predominantly children^19^ or only children^20^ that, for their characteristic anti-parasite immunity are not comparable with adult population.^16^

HIV-positive patients with malaria presented higher incidence of anaemia,^21^ as demonstrated in a study in which co-infected patients showed lower haemoglobin levels compared to subjects with only malaria;^22^ in fact, both malaria and HIV may cause anaemia, as well as some drugs (e.g. zidovudine – AZT) used for HIV treatment.^23^

Looking now at the consequences of malaria infection on HIV disease progression, several studies showed an increased HIV-RNA replication in patients with malaria. Hoffman et al found 7-fold higher levels of HIV-1 RNA in patients with malaria than in those with only HIV infection (p <0.0001)^24^ and another study showed that HIV-1 RNA concentrations were even 10-fold higher in co-infected patients in respect of HIV mono-infected patients.^5^ In a trial conducted in Malawi, Kublin and colleagues demonstrated that viral load returned at baseline levels only after 8–9 weeks of antimalarial therapy.^25^ Epidemiological implications of this aspect are that higher HIV-transmission rates are present in co-infected patients.^26^

Based on data presented, we suggest a strict monitoring of co-infected patients in order to improve the outcomes of these two infections, in particular for people most at risk of complications, such as pregnant women, in particular in stable malaria transmission areas where adults’ parasitaemia values are often below threshold of detection.

## Malaria and HIV Co-Infection in Pregnant Women

It is estimated that each year about 24 million pregnant women are infected by *P. falciparum*, especially in sub-Saharan Africa^8^ and about 1 million per year are co-infected with HIV.^27^ It is known that co-infected pregnant women are particularly at risk of complications due to these two infections; in fact, women during pregnancy are more likely susceptible to malaria disease than non-pregnant women,^28^ and a down regulation of the adaptive immune response was observed with a consequent enhanced placental invasion.^29^ High levels of parasitaemia and chronic parasite infection in placental blood can lead to consumption of nutritive blood substances,^30^ to a worsening of perinatal outcomes and to increased rates of maternal morbidity.^31^ In this context co-infection with HIV represents a further immune system impairment, which can be a concurrent cause of uneffectiveness in parasitaemia control.^12^

Consequences of this co-infection are described in both directions: HIV may promote more severe malaria clinical manifestations and, on the other hand, malaria can favours HIV RNA replication threatening antiretroviral treatment effectiveness.^32^

In co-infected pregnant women, all the adverse pregnancy outcomes are present. HIV impaired malaria outcomes inducing chronic parasitaemia, higher parasite densities and fever, reflecting in more severe clinical malaria manifestations.^12^,^33^ In a study conducted by ter Kuile and colleagues data from several works were summarized: the risk ratio [RR] of malaria parasitaemia in co-infected women were 1.58 during pregnancy, 1.66 at the time of delivery and 1.66 in the placenta in respect of HIV-uninfected women.^34^ There is also an higher risk of postpartum maternal anaemia: Ayisi et al. found that the probability to manifest this clinical condition was more than twice higher in women co-infected than in HIV-uninfected women.^35^ Malaria and HIV infections were associated with several negative outcomes in newborns to co-infected mothers. Ticconi et al. demonstrated an association between co-infection and an increased risk of stillbirth (OR = 4.74, 95% CI: 1.34–16.78) and preterm delivery (OR = 4.10, 95% CI: 2.17–7.75). The two infections, instead, resulted independently associated with an increased risk of low birth weight (malaria: OR = 10.09, 95% CI: 6.50–15.65; HIV: OR = 3.16, 95% CI: 1.80–5.54) and foetal growth retardation (malaria: OR = 3.98, 95% CI: 2.51–6.30; HIV: OR = 4.07, 95% CI: 2.40–6.92) compared to HIV-uninfected women.^36^ Low birth weight (LBW) prevalence appear to be higher in co-infected women (P=0.001) compared to HIV mono-infected women (p=0.09) and malaria infected alone (P=0.006). In addition infant mortality seems to be higher in infants born to co-infected women. A study conducted in Malawi reported significantly higher mortality rates among children born to HIV-seropositive compared to HIV-seronegative women, and in the multivariate model the risk of neonatal mortality was 4.5 greater in co-infected mother compared to mother with only placental malaria and 2.7–7.7 greater in HIV mono-infected mothers.^37^

As previously said, malaria promotes HIV RNA replication: a study showed that women had about 2-fold increase in HIV-1-RNA both in peripheral and placental blood, with a consequent higher possibility of mother-to-child transmission of HIV,^38^ even if, at present, a clear association between placental malaria and an increase of mother-to-child transmission of HIV infection still remains uncertain.^12^

An appropriate control of both infections is a priority either for women and for their infants. The main recommendations are: a prompt HAART initiation in pregnant women as soon as the eligibility criteria are met, or an effective antiretroviral prophylaxis if criteria for initiating ART are not present, according to World Health Organization (WHO) specific guidelines.^39^ Therefore a proper prophylaxis with co-trimoxazole after the first trimester of pregnancy seems to prevent both opportunistic infections and malaria.^34^

## Malaria in HIV-Infected International Travellers

According to the World Tourism Organization (WTO), an increasing number of international travellers has been reported, from 50 million/year after second World War to 980 million/year during 2011.^40^ Approximately 80 million persons from industrialized nations travel to the tropical world each year.^41^ Imported malaria mostly occurs in tourists and migrants travelling to their origin countries to visit friends and relatives (VFR)^42^ but higher rates of malaria infection have been reported also among HIV-infected people.^43^

This is mainly due to the introduction of HAART and the improved quality of life allowed a steady increase of international travels, especially to tropical areas.^44^ A recent report showed that international travel among HIV-infected people was associated with poor adherence to antiretroviral therapy, risky sexual practices and risky exposure to travel-related diseases.^45^ Although any travel-related disease may have more severe clinical manifestations in HIV positive travellers compared to HIV-uninfected persons, malaria is certainly one of the most dangerous conditions;^46^ therefore HIV-infected patients should be more aware of the necessity for medical counsel prior to travel^47^,^48^ particularly to prevent malaria infection.

Several cases of imported malaria, as well severe cases with fatal outcome, have been reported;^49^,^50^,^51^,^52^ however clinical presentation is often similar to HIV-negative patients as recently reported in a retrospective study conducted in Spain.^53^ These data suggest that more caution should be taken during the pre-travel counselling.

WHO recommends a viro-immunological parameters check (CD4+ T cell count and HIV viraemia at least) and a clinical examination before travelling.^54^ Obviously, for HIV-infected pregnant women and young children travel should be avoided if not strictly necessary as they have higher risks of severe malaria clinical manifestations.

Then, physicians involved in HIV care as well as in travel medicine should consider several factors in pre-travel counselling for HIV-infected travellers such as reason for travel (tourism, VFR, business etc.), travel risk (pre-arranged or organized travel), travel destination, season and malarial epidemic cycle as well as *Plasmodium* drug sensitivity in the specific area;^10^ moreover HIV-infected people travelling to malaria endemic-areas need to start a correct chemoprophylaxis regimen before their travel and counsellors should consider possible drug interactions between antiretroviral and antimalarial drugs.^10^

Therefore the importance of behavioral preventive measures (bed nets, repellents, *etc.*), adequate chemoprophylaxis and, in selected circumstances, stand-by emergency treatment in case of evocative symptoms should be strongly recommended.

## Effects of Co-Trimoxazole Prophylaxis and Antiretroviral Therapy on Malaria

According to major International Guidelines^55^,^56^ HAART regimens should contain at least three active drugs from two different classes, usually 2 nucleos(t)ide reverse transcriptase inhibitors (NRTIs) accompanied with an anchor drug which is either a non-nucleoside reverse transcriptase inhibitors (NNRTI) or a protease inhibitor (PI) with a low-dose of ritonavir (RTV) as “booster”. Sustained virological suppression and CD4+ T cell count recovery is the main objective of HAART.^55^ In HIV-infected patients, daily co-trimoxazole should to be prescribed as prophylaxis against major opportunistic infections when CD4+ T cells are less than 200/μl;^57^ however several findings demonstrated that daily co-trimoxazole prescription may reduce the occurrence of parasitaemia^58^ and clinical malaria either in adults and in children.^59^,^60^,^61^

For example, Mermin J and colleagues^58^ found that among HIV-infected children the use of co-trimoxazole prophylaxis and insecticide-treated nets might reduce the prevalence of clinical malaria by 97% *versus* the reduction of 43% with nets alone. Therefore co-trimoxazole prescription in immunocompromised patients, in addition at insecticide-treated nets, seems to be an effective strategy to prevent malaria. However, cross resistance between co-trimoxazole and sulfadoxine-pyrimethamine (SP) has been reported,^62^ therefore widely use of co-trimoxazole may promote specific SP resistance; some studies did not find SP related resistances despite co-trimoxazole prescription,^63^,^64^ while others found that co-trimoxazole prophylaxis was effective despite antifolate resistance.^60^

Therefore some authors suggested that a continuation of prophylaxis with co-trimoxazole in malaria endemic areas is beneficial even when patients have been immune restored.^11^ Antiretroviral therapy may be effective against malaria infection: Mermin J et al^65^ in a prospective cohort study found that HAART containing NRTIs when co-administrated with co-trimoxazole decrease malaria clinical presentation in respect of co-trimoxazole alone; in order to provide an explanation on the possible antimalarial activity of NRTIs, authors hypothesized that HAART probably reduced the frequency of malaria by improving immune function rather than by a direct antimalarial effect. A delayed *P. falciparum* clearance in HIV-infected patients treated with artemisinin-based regimens has been reported^66^ suggesting that immune defences of HIV-infected people may affect effectiveness of antimalarial treatment. Other studies evaluating impact of PIs are ongoing (e.g. NCT00719602) but available studies “in vitro” showed that the PIs lopinavir, saquinavir, indinavir, atazanavir ritonavir have direct effect on inhibition of *P. falciparum*, at concentration used in clinical practice.^67^,^68^ Anyway, antiretroviral treatment maybe effective against *P. falciparum* and may reduce malarial clinical manifestation, but, in order to better clarify this hypothesis, its underlying biological mechanisms and the role of antiretroviral drugs or classes need further investigations.

## Interactions Between Antiretrovirals and Antimalarial Agents:

### Antiretrovirals and antimalarial drugs (treatment)

Pharmacological interactions between antiretroviral and antimalarial drugs has been previously extensively reviewed,^69^,^13^ however the knowledge about this critical topic is still debated and data reported are not definitive, particularly due to either the paucity of data about newer drugs and classes of antiretroviral agents and the absence of specific guidelines. Moreover the relevance of “in vitro” studies in common clinical practice is controversial. According to WHO, first-line treatment for uncomplicated malaria in high-endemicity areas consists of a combination of either artemether plus lumefantrine or artesunate plus one of the following drugs: amodiaquine, mefloquine, or sulfadoxine–pyrimethamine, or dihydroartemisinin–piperaquine.^1^ Interactions between amodiaquine and efavirenz has been reported reflecting in higher amodiquine concentrations;^70^ these evidences may have a clinical impact, as a significant number of HIV-infected patients in sub-Saharan Africa receive an efavirenz-based treatment^57^ and WHO contraindicates this co-administration;^27^ furthermore, given that these drugs are both potentially hepatotoxic^71^,^72^ patients prescribing this combination have to be strictly monitored. Finally, we know that quinine, halofantrine, and lumefantrine are all antimalarial drugs metabolized through the cytochrome P-450 enzyme system;^73^,^74^ so that, these drugs may potentially interact with the non-nucleoside reverse transcriptase inhibitors (NNRTIs) resulting in reduced bioavailability of the antimalarial drugs.^75^ Recently, a cross-sectional study in HIV and malaria co-infected adults showed significant pharmacokinetic interactions among nevirapine, artemether and dihydroartemisinin: these interactions maybe increase the risk of treatment failure of both infections and the risk for development of drug resistance.^76^ Drug-interactions with PIs, that are also metabolized through the hepatic cytochrome P-450 enzyme system, and enzyme inhibition by ritonavir may on the contrary increase serum concentrations of these antimalarials.^77^ Despite this study, performed in healthy volunteers, ritonavir might boost the bioavailability of either PIs and antimalarial drugs, however this altered pharmacokinetics do not seems clinically relevant. By contrast, a recent study confirmed that LPV/r-based HAART significantly increases lumefantrine exposure without an increase in adverse effects. Artemether and dihydroartemisinin concentrations were also significantly increased by ritonavir boosted lopinavir-based treatment, but to a lesser extent.^78^ Data from randomized-controlled trials will be highly valuable in evaluating the clinical significance of all these interactions.

Most of potential clinically significant interactions between antimalarial and antiretrovirals drugs are showed in **Table 1**.

### Malaria chemoprophylaxis in HIV infected subjects

Despite several reports and reviews on malaria and HIV co-infection, few data are present in literature about the use of malaria chemoprophylaxis in HIV infected travellers.^57^ Mefloquine reduces some ritonavir boosted protease inhibitors levels and mefloquine plasma levels could be reduced by efavirenz and nevirapine, but these data are from patients who received mefloquine as treatment, little is known about its use in chemoprophylactic regimen. Atovaquone/proguanil levels could be reduced by indinavir, as well as lopinavir, atazanavir, ritonavir and efavirenz;^79^ moreover, atovaquone/proguanil could reduce indinavir levels and increase zidovudine levels causing hematological troubles.^80^ No significant data are available on doxycycline interactions.^81^ All these considerations should be taken into account before starting chemoprophylaxis in HIV infected travelers taking HAART, and drugs dosages should be arranged in order to avoid drug interactions. If chemoprophylaxis is not administered, atovaquone/proguanil might be a suitable *stand-by emergency treatment* (SBET) option for HIV-infected subjects receiving NNRTI-based regimens, while mefloquine could be considered as SBET drug in those prescribing PI-based HAART.^82^

## Conclusion And Future Research Perspectives

Malaria and HIV infections are two of the most important infectious diseases worldwide, particularly in sub-Saharan Africa: their overlapping epidemiology as well their impact in clinical practice needs to be continuously updated.

HIV-infected adults, especially if international travelers, even with a restored immunity, are more likely to have severe clinical manifestations of malaria compared to travellers without HIV infection. Moreover co-infection in pregnant women contributes to develop clinical malaria in women, maternal severe anaemia, grater rates of stillbirth and preterm delivery, low birth weight, foetal grow retardation and increased risk of infant mortality.

The wider implementation of co-trimoxazole prophylaxis and antiretroviral therapy could change the impact of malaria in HIV-infected patients.

The benefits of the continuation of co-trimoxazole prophylaxis to prevent malaria, the antimalarial effects of several antiretroviral agents, as well the interactions between antiretroviral and antimalarial drugs, need to be further investigated. The proper malaria prophylaxis and treatment in HIV-infected pregnant women also needs to be defined. Several clinical trials are ongoing to address several of these opened questions: the results of these trials are needed to create specific guidelines for the prevention and management of malaria and HIV co-infection and to help clinicians involved in travel medicine as well in HIV care.

## Figures and Tables

**Table 1:** Potential interactions between antimalarial and antiretroviral drugs.

**Antimalarial drugs**	**Antiretroviral drugs**	**Potential interactions**
**Quinine**	EFV, NVP	**Reduced quinine concentrations**
**Quinine**	PI	**Reduced quinine concentrations**
**Atovaquone/Proguanil**	RTV, LPV/r, ATV/r, IDV, EFV	**Reduced atovaquone/proguanil concentrations**
**Atovaquone/Proguanil**	IDV	**Reduced IDV concentrations**
**Atovaquone/Proguanil**	AZT	**Increased AZT concentrations** **Increased risk for anaemia**
**Mefloquine**	RTV	**Reduced RTV concentrations**
**Mefloquine**	EFV, NVP	**Reduced mefloquine concentrations**
**Lumefantrine**	PI	**Increased lumefantrine concentrations** **Prolongation of QT interval**
**Lumefantrine**	NVP, EFV, DLV	**Reduced lumefantrine concentrations** **Prolongation of QT interval**
**Halofantrine**	PI	**Increased halofantrine concentrations** **Prolongation of QT interval**
**Halofantrine**	NVP, EFV, DLV	**Reduced halofantrine concentrations** **Prolongation of QT interval**
**Amodiaquine + artesunate**	EFV	**Increased amodiaquine concentrations** **Increased transaminase levels**
**Chloroquine**	RTV	**Alteration of antimalarial drug metabolism**
**Pyrimethamine**	RTV	**Alteration of antimalarial drug metabolism**
**Sulfadoxine-pyrimethamine**	RTV	**Alteration of antimalarial drug metabolism**
**Sulfadoxine-pyrimethamine**	NVP	**Possible adverse cutaneous or hepatic reactions**
**Sulfadoxine-pyrimethamine**	AZT	**Increased risk for anaemia**
**Sulfadoxine-pyrimethamine**	Co-trimoxazole	**Increased risk of severe adverse cutaneous or hepatic reactions**
**Artemisinin**	PI	**Alteration of artemisinin metabolism**
**Dapsone**	SQV	**Alteration of dapsone metabolism**

Modified by Flateau C, Lancet Infect Dis, 2011; Skinner-Adams TS, Trends Parasitol, 2008; Khoo S, AIDS, 2005.^11^,^13^,^69^

EFV: efavirenz; NVP: nevirapine; PI: protease inhibitors; RTV: ritonavir; LPV: lopinavir boosted by ritonavir; ATV/r: atazanavir boosted by ritonavir; IDV: indinavir; AZT: zidovudine; DLV: delavirdine; SQV: saquinavir.
